# Citrullination only infrequently impacts peptide binding to HLA class II MHC

**DOI:** 10.1371/journal.pone.0177140

**Published:** 2017-05-08

**Authors:** John Sidney, Stephane Becart, Mimi Zhou, Karen Duffy, Mikaela Lindvall, Erin C. Moore, Eugene L. Moore, Tadimeti Rao, Navin Rao, Morten Nielsen, Bjoern Peters, Alessandro Sette

**Affiliations:** 1 La Jolla Institute for Allergy and Immunology, La Jolla, California, United States of America; 2 Janssen Research & Development, San Diego, California, United States of America; 3 Center for Biological Sequence Analysis, Department of Bio and Health Informatics, The Technical University of Denmark, Lyngby, Denmark; 4 Instituto de Investigaciones Biotecnológicas, Universidad Nacional de San Martín, San Martín, Buenos Aires, Argentina; Hospital Israelita Albert Einstein, BRAZIL

## Abstract

It has been hypothesized that HLA class II alleles associated with rheumatoid arthritis (RA) preferentially present self-antigens altered by post-translational modification, such as citrullination. To understand the role of citrullination we tested four RA-associated citrullinated epitopes and their corresponding wild-type version for binding to 28 common HLA class II. Binding patterns were variable, and no consistent impact of citrullination was identified. Indeed, in one case citrullination significantly increased binding compared to the WT peptide, in another citrullination was associated with a reduction in promiscuity by 40%. For a more comprehensive analysis, we tested over 200 citrullinated peptides derived from vimentin and collagen II for their capacity to bind the RA-associated shared epitope alleles DRB1*01:01 and DRB1*04:01. The overall effect of citrullination on binding was found to be relatively minor, and only rarely associated with 3-fold increases or decreases in affinity. Previous studies have suggested that citrullination of MHC anchor residues, in particular P4, is associated with generation of novel RA-associated epitopes. However, analysis of the predicted MHC-binding cores of all peptides tested found that in modified peptides with increased binding affinity the citrullinated residue was predicted to occupy an anchor position in only a minority of cases. Finally, we also show that identification of citrullinated peptide binders could be facilitated by using the NetMHCIIpan 3.1 algorithm, representing citrullination as a wildcard. Our studies identify a total of 117 citrullinated peptides that bound RA-associated alleles with an affinity of 1000 nM or better.

## Introduction

The HLA-DRB1 locus is associated with rheumatoid arthritis (RA) in most racial groups and accounts for approximately one-third of the genetic susceptibility to RA [1, 2]. The Shared Epitope (SE) is a conserved amino acid sequence at positions 70–74 in the third hypervariable region of the DRB1 chain shared between RA-associated alleles (e.g., DRB1*04:01 and DRB1*01:01) and contributes to the P4 peptide-binding pocket. The presence of SE is the single most significant genetic risk factor for RA [3]. It has been shown that 65–85% of RA patients carry at least one MHC class II risk allele (i.e., SE+) [4–7], as opposed to protective alleles (SE-), such as HLA-DRB1*04:02 [3, 8]. The SE not only confers a higher risk for RA, but also increases the likelihood of developing a more severe form of the disease [9]. SE-coding HLA-DRB1 alleles are associated with earlier onset of arthritis and more severe bone erosion [10]. Furthermore, there is evidence of a gene-dose effect, in which the severity of bone destruction in RA correlates positively with the number of SE-coding HLA-DRB1 alleles [7, 11].

Over the past decade, a central role has been recognized for post-translational modifications, with particular emphasis on citrullination, in the pathogenesis of RA [12, 13]. Citrullination is a post-translational modification of arginine by peptidylarginine deiminase (PAD) enzymes and is increased in response to stress/inflammation. This modification results in a loss of net positive charge, and has been proposed to increase the peptide binding affinity of citrullinated peptides to SE+ alleles [14]. In the context of autoimmune disease, citrullination may thus promote generation of high-affinity citrullinated (and not arginine-containing) neo-autoantigens, triggering activation of autoreactive T and B cells and inducing an autoimmune response. By contrast, protective HLA molecules may be able to bind both arginine and citrulline, leading to negative selection of the recognizing T cells, thereby preserving tolerance to these autoantigens. Supporting this hypothesis, it has been shown that (i) citrullinated fibrinogen could induce arthritis in DRB1*04:01 transgenic mice [15], (ii) proinflammatory cytokines were produced by CD4+ T cells in SE+ individuals in response to citrullinated self-epitopes [16], (ii) citrulline-specific Th1 and Th17 cells are increased in HLA-DRB1*04:01^+^ RA patients [17, 18], and (iii) RA patients display Th17 cells responding to arthritogenic citrullinated aggrecan [19]. Furthermore, Anti-Citrullinated Protein Antibodies (ACPA) recognizing various citrullinated antigens such as fibrinogen, vimentin or collagen type II, are highly specific for RA and are detected in approximately 70% of RA patient sera [20–23]. ACPA-positive individuals have the highest risk of RA [24–27]. Indeed, ACPA-positivity was found to be associated with a 10-fold increased risk overall, and a 33-fold increased risk of RA onset within 5 years [28]. Moreover, the presence of ACPA is also predictive of a more severe disease course [29–31] and it has been further shown that passive transfer of ACPA in mice with existing minimal joint disease exacerbates disease [32] and promotes bone destruction [33]. In addition, the individuals harboring both risk HLA-DR SE-positive alleles and any ACPA have the highest risk for RA and more severe progression of the disease [31]. Importantly, ACPAs emerge before onset of disease (up to 14 years prior to disease) and show a marked increase about 2 years prior to RA diagnosis [28, 34]. This offers an opportunity for early interception in a stratified patient subset (SE+, ACPA+ asymptomatic “patients”) and underlines the need to better understand the impact of citrullination on autoimmune responses.

It has been hypothesized that RA-predisposing HLA-DR SE+ alleles bind arthritogenic peptides whose affinity is increased by posttranslational modification (e.g., citrullination) [14]. Studies by Scally *et al*. [35] and others [14] proposed that while both predisposing and protective alleles bind citrullinated peptides, only the protective alleles bind the native peptide. It has also been hypothesized that autoimmune epitopes are associated with unique binding properties and lower affinities or different patterns of specificity [36], and that citrullination might impart unique binding properties to modified peptides, underlying the development of autoantigen specific T cell responses.

To experimentally address these points, we measured binding affinities of native and citrullinated versions of several arthritogenic peptides using an *in vitro* assay and a panel of 28 widely expressed HLA class II purified molecules, with a particular focus on SE- vs SE+ HLA-DR alleles. We also tested a large panel citrulline modified (cit) peptides derived from vimentin and collagen II, and their corresponding WT version, for their capacity to bind the SE+ alleles DRB1*01:01 and DRB1*04:01. Together, these studies revealed that in general the effect of citrullination on binding is minor, with the vast majority of cases resulting in little positive or negative difference in binding affinity. Further, after determining the specific frames in which each peptide bound the SE+ DRB1*01:01 and DRB1*04:01 alleles, we found that increases in binding could more often be attributed to modification of non-anchor residues. Finally, we demonstrate that the use of HLA class II binding algorithms representing citrullinated amino acids as wildtype (“X”), when combined with *in vitro* binding assays, are an efficient means to predict and identify potential citrullinated epitopes.

## Methods

### MHC purification and peptide binding studies

Purification of HLA class II MHC molecules by affinity chromatography, and the performance of assays based on the inhibition of binding of a high affinity radiolabeled peptide to quantitatively measure peptide binding, were performed essentially as detailed elsewhere [37]. Briefly, EBV transformed homozygous cell lines were used as sources of MHC molecules. A high affinity radiolabeled peptide (0.1–1 nM) was co-incubated at room temperature or 37°C with purified MHC in the presence of a cocktail of protease inhibitors. Following a two-day incubation, MHC bound radioactivity was determined by capturing MHC/peptide complexes on mAb (HLA-DR: L243; HLA-DQ: SPVL3; HLA-DP: B7/21) coated Lumitrac 600 plates (Greiner Bio-one, Frickenhausen, Germany), and measuring bound cpm using the TopCount (Packard Instrument Co., Meriden, CT) microscintillation counter. The concentration of peptide yielding 50% inhibition of the binding of the radiolabeled peptide was calculated. Under the conditions utilized, where [label]<[MHC] and IC_50_ ≥ [MHC], the measured IC_50_ values are reasonable approximations of the true Kd values [38, 39]. Each competitor peptide was tested at six different concentrations covering a 100,000-fold range, and in three or more independent experiments. As a positive control, the unlabeled version of the radiolabeled probe was also tested in each experiment. Peptides were purchased from Mimotopes (Victoria, Australia) and/or A and A (San Diego) as crude material on a 1 mg scale, or purified (>95%) by reverse phase HPLC. Binding predictions, and prediction of peptide binding cores, were performed using the NetMHCIIpan-3.1 algorithm (www.cbs.dtu.dk) [40].

### Selection of a panel of 28 common class HLA II molecules

For HLA-peptide binding studies, we selected a panel of HLA class II molecules (S1 Table) representing common specificities in the general population [41], as well as a range of SE/non-SE associated motifs [42]. The panel included several HLA-DRB1 “protective alleles” associated with non-SE motifs, and found to have Odds Ratio (OR) values less than 1 (see [42]). These protective alleles included 3 HLA-DRB1 alleles (*0402, *1302 and *1501) associated with the S1 motif (ARAA or ERAA; OR = 0.39) and 3 (*1101, 1201 and 1602) with the S3D motif (DRRAA; OR = 0.37). We further included four “neutral” HLA-DRB1 alleles (*0301, *0701, *0802 and *0901) that do not have a specific SE motif, and that are associated with OR = 1.00. Finally, we selected 5 HLA “predisposing” alleles, to include four (*0101, *0404, *0405 and *1001) with the S3P motif (Q/R RRAA; OR = 1.61), and one (*0401) with the S2 motif (KRAA; OR = 3.21). Four to five allelic variants each were also selected to represent the HLA-DRB3/4/5, HLA-DQ and HLA-DP loci. These molecules are not particularly associated with RA or SE, but are nevertheless expressed alongside HLA-DRB1 alleles. The phenotypic frequency at which each molecule is expressed in the general population is also shown in S1 Table. Population coverage was calculated as previously described [41, 43]. Gene frequencies (gf) for each HLA allele were calculated from population frequencies obtained from DbMHC (NCBI; [44]). Phenotypic frequencies (pf) were calculated utilizing the binomial distribution formula: pf = 1 − (1 − ∑gf)^2^, assuming no linkage disequilibrium.

## Results

### HLA binding affinity and specificity of RA-associated epitopes

To investigate the effects of citrullination on HLA class II binding, we first selected a panel of T cell epitopes previously described as potentially arthritogenic when citrullinated (Table 1), including fibrinogen 78–91 [15, 16], aggrecan 84–103 [16, 19, 45, 46], vimentin 66–78 [17, 18] and collagen II 1236–1249 [16]. For each, we tested both the wild-type (WT) and citrullinated (cit) versions for their capacity to bind a panel of 28 common HLA class II [41], including a range of SE/non-SE associated variants [42] (see S1 Table). To benchmark affinity and specificity measures, we also tested the promiscuous influenza HA 307–319 T cell epitope [47, 48].

**Table 1:** HLA class II binding of RA-associated epitopes^1^.

Group	Allele	HA 307–319	Fibrinogen 78–91		Aggrecan 84–103		Vimentin 66–78		Collagen II 1236–1249	
		WT^2^	WT	Cit	WT	Cit	WT	Cit	WT	Cit
SE+	DRB1*01:01	**4.2**	-	15655	**155**	3863	3256	**9.5**	**81**	**232**
	DRB1*04:01	**33**	-	12878	**713**	**827**	13109	**1.8**	**9.7**	**13**
	DRB1*04:04	**751**	2049	2323	**565**	**854**	**206**	**9.0**	**102**	**216**
	DRB1*04:05	**59**	12770	11260	**183**	**47**	3957	**54**	1766	7343
	DRB1*10:01	**206**	3578	8484	**643**	2416	5167	**35**	**375**	**432**
SE-	DRB1*04:02	**12**	-	-	**501**	4264	**927**	**14**	**118**	**211**
	DRB1*11:01	**126**	13892	-	**520**	29652	11831	8924	9855	-
	DRB1*12:01	1233	27769	9377	1048	11024	15874	2220	-	-
	DRB1*13:02	**45**	-	-	**268**	**552**	11442	15033	3915	8288
	DRB1*15:01	**797**	-	-	1007	7600	**4.1**	**5.4**	1457	15165
	DRB1*16:02	**350**	8432	15268	20460	22841	1080	**118**	23114	-
Other DR	DRB1*03:01	4257	-	-	**530**	11145	12207	25930	**159**	**72**
	DRB1*07:01	**385**	-	-	3034	8475	-	7306	29581	-
	DRB1*08:02	1082	**457**	35746	3959	16957	**53**	**275**	1037	2148
	DRB1*09:01	**824**	11063	-	**730**	28476	1415	**353**	**315**	**276**
	DRB3*01:01	4309	-	-	8464	18930	-	-	**68**	**63**
	DRB3*02:02	**456**	-	-	**245**	**51**	9086	-	**262**	**481**
	DRB4*01:01	**462**	7696	12685	**955**	2341	2023	**4.0**	**80**	**7.3**
	DRB5*01:01	**75**	**23**	**9.2**	**16**	**740**	2228	1360	8880	10187
DP	DPB1*02:01	24753	-	-	14833	2768	-	-	-	-
	DPB1*04:01	39067	-	-	26153	16155	-	-	-	-
	DPB1*04:02	1900	14564	23530	2143	13264	-	-	-	-
	DPB1*05:01	15236	16209	20823	7043	19539	9411	11946	-	-
DQ	DQB1*02:01	4786	-	-	2542	**783**	-	-	**413**	**198**
	DQB1*03:01	19944	-	-	**229**	**382**	2095	2009	12781	4118
	DQB1*03:02	-	-	-	2444	4122	-	-	-	-
	DQB1*05:01	37206	-	37424	2641	7117	-	-	-	10320
	DQB1*06:02	**389**	-	-	**630**	**513**	**11**	**25**	1342	1525
Alleles bound	SE+	5	0	0	5	3	1	5	4	4
	SE-	5	0	0	3	1	2	3	1	1
	Other DR	5	2	1	5	2	1	3	5	5
	DP	0	0	0	0	0	0	0	0	0
	DQ	1	0	0	2	3	1	1	1	1
	Total	16	2	1	15	9	5	12	11	11

^1.^ Epitopes sequences, where X indicates the position of citrullinated arginine: HA 307–319, PKYVKQNTLKLAT; Fibrinogen 78–91, NQDFTNXINKLKNS; Aggrecan 84–103, VVLLVATEGXVRVNSAYQDK; Vimentin 66–78, SAVRLXSSVPGVR; Collagen II 1236–1249, LQYMXADQAAGGLR.

^2.^ Binding expressed as IC50 nM, and a binder (highlighted with bold font) is defined as IC50 <1000 nM. A dash (-) indicates IC50 >30,000 nM.

Confirming previous reports, HA 307–319 bound 15/19 HLA-DR alleles tested (but only 1/9 non-DR HLA class II) with an IC_50_ <1000 nM (Table 1), including all 5 SE+ alleles and 5/6 SE- alleles. Also shown in Table 1, the citrullinated version of fibrinogen 78–91 bound only one of the 28 alleles, and the unmodified WT version bound just two. Thus, citrullination has little effect on binding of this non-promiscuous HLA class II binding epitope. Aggrecan 84–103 [16, 19, 49], on the other hand, was relatively promiscuous, binding a total of 15/28 HLA tested including all 5 SE+, and 3/6 SE-, alleles. By contrast, the citrullinated version bound only 9 alleles and lost binding to 4 alleles, including 2 SE+ alleles (DRB1*01:01 and DRB1*10:01). Thus, citrullination has an indiscriminate negative effect on this promiscuous binder.

Vimentin 66–78, in both WT and citrullinated forms, has been described as DRB1*04:01-restricted [17]. The WT peptide bound only 5/28 HLA tested (Table 1), while the citrullinated version bound of 12/28 alleles, including all 5 predisposing alleles and 3/6 protective alleles. Increases in binding with citrullination were in most cases over 20-fold, and in several cases >100-fold. Thus, citrullination of the vimentin epitope increased binding capacity, in particular for RA predisposing specificities. By contrast, both the WT and citrullinated versions of collagen II 1236–1249, which is associated with T cell responses of undefined restriction [16] bound 11 of the 28 HLA tested, including 4/5 SE+ alleles and 1/6 SE- alleles, with almost identical affinities.

Taken together, this data shows that RA-associated epitopes in their WT and citrullinated forms have variable binding patterns to HLA class II alleles, and a consistent impact of citrullination is not apparent. That is, some citrullinated peptides bind better, some bind the same, and others bind worse, than their cognate wild-type sequence.

### Position of citrullination and influence on binding capacity of known epitopes

Previous studies have suggested that citrullination has an impact when it occurs at an anchor position, and in particular P4. This observation, along with the data above, suggests that citrullination may generate novel epitopes in two ways. First, a new epitope may be generated through substantially increased MHC class II binding capacity by modification at an MHC contact residue (i.e., an anchor residue). Alternatively, a new epitope by be generated through modification at a non-anchor residue that would be available for contact with a specific TCR.

To see if these patterns might be reflected in the above data, we utilized the NetMHCIIpan 3.1 tool (version 3.1, as hosted at www.cbs.dtu.dk) [40] to predict the peptide-binding core for each of the four T cell epitopes tested above. For the present analysis, we focused on the 15 DRB1 alleles, for which a canonical P1-P4-P6-P9 anchor spacing has been generally described [50, 51]. For each epitope and allele, we predicted the core for each WT epitope, and determined the relative position of citrullination.

As shown in Table 2, in only 11 of 60 cases (18.3%) did citrullination result in a 3-fold or greater increase in binding capacity. In 35/60 (58.3%) cases the effect of citrullination on binding was considered neutral, and in the remaining 14 (23.3%) negative. Citrullination was predicted to be in P4 in 24/60 cases (40%), and P4 citrullination accounts for 8 of the 11 cases (72.7%) where binding was increased. However, examining the data on an epitope-by-epitope basis revealed that the vimentin 66–78 epitope accounts for 10 of the 11 cases where improved binding was associated with citrullination of the P4 residue. For fibrinogen, by contrast, where for 14/15 alleles examined citrullination did not result in improved binding, the citrullination was also predicted to be in P4. At the same time, for collagen II, where the binding of the WT and citrullinated peptides were largely identical, in all cases the citrullination of the non-anchor P2 and P3 residues was predicted. Finally, for aggrecan, where citrullination has a deleterious influence on binding for 8 DRB1 alleles, in 4 cases the citrullination was in the P6 anchor position, but deleterious influences were also noted for citrullination at the P5 and N-1 non-anchor positions, and the P7 secondary anchor [50].

**Table 2:** Predicted HLA DR binding cores of four known citrullinated RA-associated epitopes.

Epitope	Sequence	Pos of cit	Allele	Effect of cit on binding^1^	Predicted WT core		
					Sequence	Pos of cit
Fibrinogen 78–91	NQDFTN**R**INKLKNS	7	DRB1*01:01	Neutral	FTNRINKLK	4
			DRB1*04:01	Neutral	FTNRINKLK	4
			DRB1*04:04	Neutral	FTNRINKLK	4
			DRB1*04:05	Neutral	FTNRINKLK	4
			DRB1*10:01	Neutral	FTNRINKLK	4
			DRB1*04:02	Neutral	DFTNRINKL	5
			DRB1*11:01	Neutral	FTNRINKLK	4
			DRB1*12:01	Neutral	FTNRINKLK	4
			DRB1*13:02	Neutral	DFTNRINKL	5
			DRB1*15:01	Neutral	FTNRINKLK	4
			DRB1*16:02	Neutral	FTNRINKLK	4
			DRB1*03:01	Neutral	DFTNRINKL	4
			DRB1*07:01	Neutral	FTNRINKLK	4
			DRB1*08:02	Decreased	FTNRINKLK	4
			DRB1*09:01	Neutral	FTNRINKLK	4
Aggrecan 84–103	VVLLVATEG**R**VRVNSAYQDK	10	DRB1*01:01	Decreased	LVATEGRVR	7
			DRB1*04:01	Neutral	LVATEGRVR	6
			DRB1*04:04	Neutral	VLLVATEGR	9
			DRB1*04:05	**Increased**	**VLLVATEGR**	**8**
			DRB1*10:01	Decreased	LVATEGRVR	6
			DRB1*04:02	Decreased	VRVNSAYQD	-1
			DRB1*11:01	Decreased	LVATEGRVR	6
			DRB1*12:01	Decreased	LLVATEGRV	7
			DRB1*13:02	Neutral	VRVNSAYQD	-1
			DRB1*15:01	Decreased	LVATEGRVR	7
			DRB1*16:02	Neutral	LVATEGRVR	7
			DRB1*03:01	Decreased	VATEGRVRV	5
			DRB1*07:01	Neutral	LVATEGRVR	7
			DRB1*08:02	Decreased	LVATEGRVR	6
			DRB1*09:01	Decreased	LVATEGRVR	6
Vimentin 66–78	SAVRL**R**SSVPGVR	6	DRB1*01:01	**Increased**	**VRLRSSVPG**	**4**
			DRB1*04:01	**Increased**	**VRLRSSVPG**	**4**
			DRB1*04:04	**Increased**	**VRLRSSVPG**	**4**
			DRB1*04:05	**Increased**	**VRLRSSVPG**	**4**
			DRB1*10:01	**Increased**	**VRLRSSVPG**	**4**
			DRB1*04:02	**Increased**	**VRLRSSVPG**	**4**
			DRB1*11:01	Neutral	VRLRSSVPG	4
			DRB1*12:01	**Increased**	**VRLRSSVPG**	**4**
			DRB1*13:02	Neutral	LRSSVPGVR	1
			DRB1*15:01	Neutral	VRLRSSVPG	4
			DRB1*16:02	**Increased**	**VRLRSSVPG**	**4**
			DRB1*03:01	Neutral	LRSSVPGVR	2
			DRB1*07:01	**Increased**	**LRSSVPGVR**	**1**
			DRB1*08:02	Decreased	VRLRSSVPG	4
			DRB1*09:01	**Increased**	**LRSSVPGVR**	**1**
Collagen II 1236–1249	LQYM**R**ADQAAGGLR	5	DRB1*01:01	Neutral	YMRADQAAG	2
			DRB1*04:01	Neutral	MRADQAAGG	2
			DRB1*04:04	Neutral	MRADQAAGG	2
			DRB1*04:05	Decreased	YMRADQAAG	3
			DRB1*10:01	Neutral	YMRADQAAG	2
			DRB1*04:02	Neutral	MRADQAAGG	2
			DRB1*11:01	Decreased	YMRADQAAG	3
			DRB1*12:01	Neutral	YMRADQAAG	2
			DRB1*13:02	Neutral	MRADQAAGG	2
			DRB1*15:01	Decreased	YMRADQAAG	2
			DRB1*16:02	Neutral	YMRADQAAG	3
			DRB1*03:01	Neutral	MRADQAAGG	2
			DRB1*07:01	Neutral	YMRADQAAG	3
			DRB1*08:02	Neutral	YMRADQAAG	3
			DRB1*09:01	Neutral	YMRADQAAG	3

^1.^ When the binding capacity of both the WT and cit versions were >10,000 nM, effect on binding was considered neutral.

The analyses in this section confirm previous observations that citrullination at the P4 position may generate peptides with much improved HLA DRB1 MHC class II binding capacity, but it is also evident that in other cases citrullination at P4 has no effect. Citrullination at other positions, both anchor and non-anchor, has a largely neutral or, less often, deleterious effect on binding capacity. These observations support the suggestion that generation of citrullinated epitopes may be the result of either increased MHC class II binding or TCR recognition.

### Effect of citrullination on binding affinity to two SE+ alleles is generally minor

Next, we sought to extend the analysis to a larger set of unbiased peptides. Accordingly, we generated a set of 15-mer peptides, overlapping by 10 residues, to span the entire protein sequence of vimentin (acc. No. P08670). Each of the 76 peptides containing an endogenous R residue was synthesized as well as analog peptides representing R to citrulline substitutions. In cases where the native peptide contained multiple R residues, multiple analogs were made, each representing a single unique citrulline substitution; a total of 127 citrullinated analogs were synthesized. The entire panel of 203 modified and unmodified peptides was then tested for binding to purified HLA-DRB1*01:01 and HLA-DRB1*04:01. We focused on these two alleles because both are SE+, and their binding specificities have been well characterized in the literature. The binding data for each peptide tested is summarized in S2 Table.

**Fig 1:**
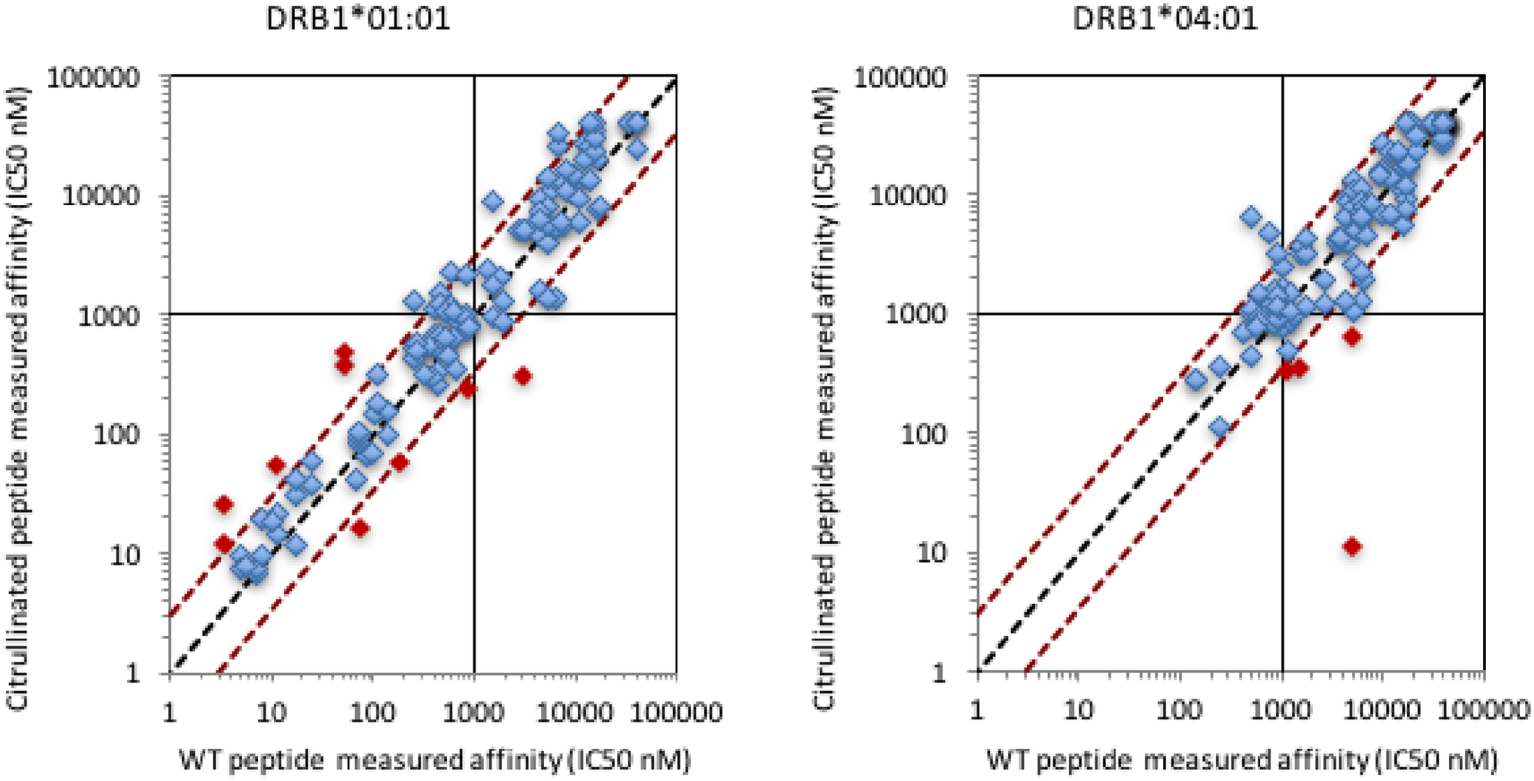
Comparison of binding affinity of wild-type and citrullinated versions of vimentin peptides. Each data point indicates the DRB1*01:01 (left panel) or DRB1*04:01 (right panel) binding capacity of WT vimentin peptides with the corresponding citrullinated version. Effects greater or less than 3-fold are demarcated by the diagonal dashed red lines and highlighted by red fill. Points to the lower right indicate instances where the citrullinated peptide binds with higher affinity that the WT peptide, and vice versa.

Fig 1 shows the HLA-DRB1*01:01 and HLA-DRB1*04:01 binding capacity of unmodified wild type (WT) peptides versus the corresponding citrullinated analogs. In only 7 (5.5%) of the 127 cases for HLA-DRB1*01:01, and 8 (6.3%) for HLA-DRB1*04:01, did a citrullinated analog bind with 3-fold or higher affinity than the corresponding WT peptide. Similarly, in only 13 (10.2%) cases for HLA-DRB1*01:01, and 3 (2.4%) for HLA-DRB1*04:01, did a citrullinated analog bind with at least 3-fold lower affinity than the corresponding WT peptide. Considered together, in 223 (87.8%) of the 254 cases considered binding capacity of the citrullinated peptide was similar to that of the corresponding wildtype peptide.

Thus, as was the case with the known epitopes, in the majority of cases in an unbiased set of peptides the effect of citrullination on HLA class II binding are minor (neutral). The analysis also identified citrullinated vimentin peptides that bound with an affinity of at least 1000 nM (a threshold previously associated with immunogenicity [50, 52, 53]) to either DRB1*01:01 (65 different epitopes), or DRB1*04:01 (22 epitopes). Of these, only 5 (7.7%) in the case of DRB1*01:01, and 4 (18.2%) in the case of DRB1*04:01, bound with 3-fold or higher affinity than the corresponding wildtype peptide.

### Predicting citrullinated binders

Based on these results, showing relatively rare and minor differences between the citrullinated and WT versions, we reasoned that existing predictive algorithms might be effectively employed to predict citrullinated binders, and hence candidate epitopes. To test this hypothesis, we predicted the HLA-DRB1*01:01 and HLA-DRB1*04:01 binding capacity of each WT vimentin peptides tested above using the stand-alone version (v3.1) of the NetMHCIIpan algorithm (as hosted at www.cbs.dtu.dk). For both alleles, the NetMHCIIpan algorithm was reasonably efficient at predicting binding of the citrullinated peptide based on scores for the WT peptide. For HLA-DRB1*01:01 a Spearman’s rank correlation (SCC) of 0.78, and an AUC (area-under-the-curve) value of 0.90, was calculated using the binding classification threshold of 1000 nM, and for HLA-DRB1*04:01 the SCC and AUC performance metrics were 0.67 and 0.83, respectively (data not shown).

**Fig 2:**
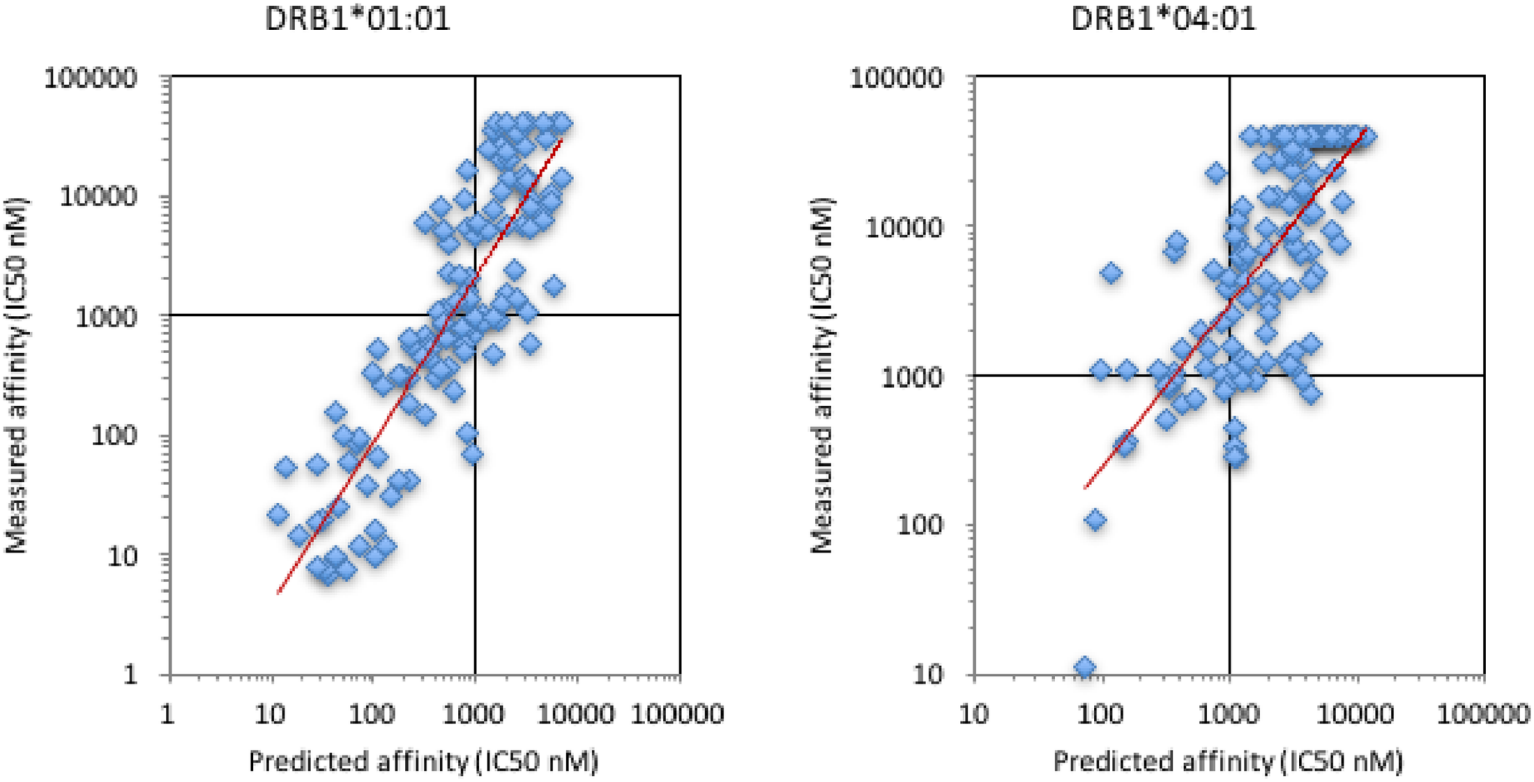
Prediction of the DRB1*01:01 and DRB1*04:01 binding capacity of citrullinated peptides. The DRB1*01:01 (left panel) and DRB1*04:01 (right panel) binding capacity of citrullinated peptides were predicted using NetMHCIIpan version 3.1 predictions by substituting the citrullinated residues with the wildcard “X”. Trend lines are show in red.

To see if improved predictions could be obtained, we next employed a feature of the NetMHCIIpan algorithm (version 3.1) that allows making predictions for peptides containing wildcard amino acids (represented as an “X”). Using this property, we evaluated the predictive performance by comparing the resulting predicted binding of the citrullinated peptides with measured binding values (Fig 2). Doing this, we found improved and relatively high performance values, with SCC = 0.84/0.70 and AUC = 0.91/0.82 for DRB1*01:01 and DRB1*04:01, respectively.

### Combination of HLA binding predictions for wild-type peptides and HLA binding assays of citrullinated analogs identifies novel citrullinated binders from collagen

To further evaluate the effectiveness of utilizing the NetMHCIIpan-3.1-based approach allowing representation of citrullinated amino acids as wildcards to predict citrullinated analogs that bind with high affinity, we examined an independent set of peptides. Accordingly, we scored all R containing 15-mer peptides in collagen using NetMHCIIpan-3.1, single substituting native R residues with X. All citrullinated peptides predicted to bind HLA-DRB1*01:01 and/or HLA-DRB1*04:01 at the 1000 nM threshold [50, 52, 53]) were selected for synthesis; in each case the corresponding WT peptide was also selected. As a result, 36 citrullinated peptides and 29 corresponding WT versions were selected for HLA-DRB1*01:01, and 13 citrullinated and 10 corresponding WT peptides were selected for HLA-DRB1*04:01. (All peptides predicted to bind DRB1*04:01 were also predicted to bind DRB1*01:01, a result that comports with previous observations regarding the broad repertoire of DRB1*01:01 [41, 54, 55]). We also selected a control set of 43 citrullinated peptides (and the corresponding 32 WT peptides) predicted to be HLA-DRB1*01:01 and HLA-DRB1*04:01 non-binders (i.e., predicted affinity >1000 nM).

These 140 collagen peptides (79 citrullinated, 61 WT) were tested for their capacity to bind HLA-DRB1*01:01 and HLA-DRB1*04:01 (S2 Table). In the case of HLA-DRB1*01:01, 35 of the 36 (97.2%) predicted citrullinated binders had an affinity of 1000 nM, or stronger. Seven of 36 citrullinated peptides also bound with an affinity at least 3-fold stronger than the corresponding WT peptide. By contrast, only 9/43 (20.9%) of the predicted non-binders bound DRB1*01:01 at the same level (8/9 had predicted affinities in the 1000–5000 nM range). For HLA-DRB1*04:01, 9 of the 13 (69.2%) of the predicted citrullinated binders had an affinity of 1000 nM or better, and 3 also bound with at least 3-fold stronger affinity than the corresponding WT peptide. For the control peptides, 4/43 (9.4%) bound DRB1*04:01 at the 1000 nM level (all 4 had predicted affinities in the 1000–2000 nM range).

**Fig 3:**
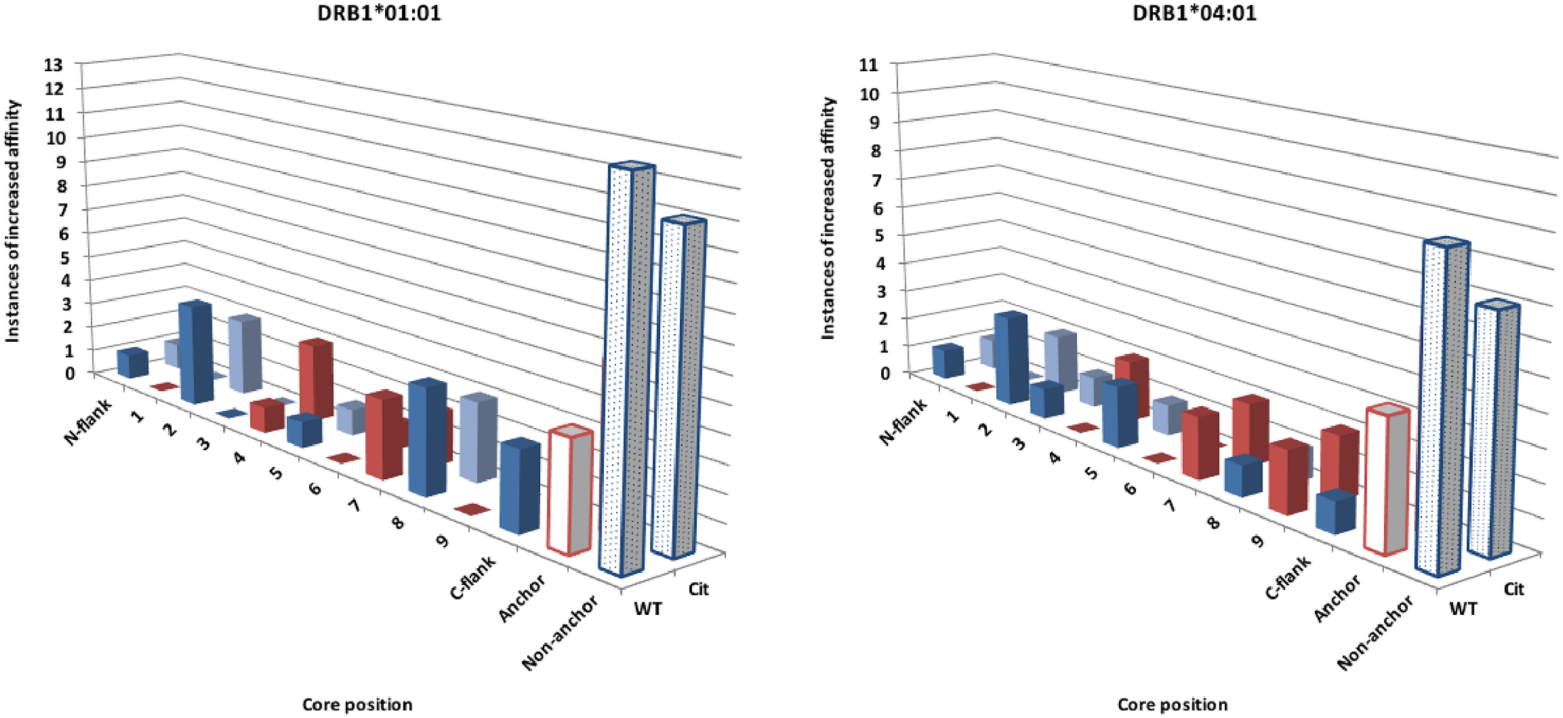
Increases in binding capacity due to citrullination is associated with modification at both anchor and non-anchor positions. The number of instances of increased DRB1*01:01 (left panel) or DRB1*04:01 (right panel) binding associated with citrullination of arginine at specific peptide positions relative to predicted core region frames is shown. Anchor positions are highlighted with red bars and non-anchor by blue bars. Light filled bars show the total number of increases attributed to anchor (white filled red bars) and non-anchor (blue hatched bars) positions. Tabulations are shown for two different approaches to defining the core residues, as described in the text.

Finally, we again utilized the NetMHCIIpan tool (version 3.1) [40] to predict the MHC binding cores of all of the citrullinated vimentin and collagen peptides tested, and the location of the corresponding citrullination was determined (see S2 Table). As above, anchor positions were defined as P1, P4, P6, P7 and P9. In 17 of the 206 (8.3%) cases for DRB1*01:01, and 13/206 (6.3%) for DRB1*04:01 (S3 Table), citrullination resulted in an increase in binding. In just 4 of the 17 (23.5%) cases improved binding for DRB1*01:01, and 4/13 (30.8%) for DRB1*0401, citrullination was predicted to be an anchor position. We also considered the possibility that with citrullination the binding core shifts frame, and as a result situates the citrullinated residue in a more optimal position (e.g., P4); this possibility is suggested as with the WT core-based predictions none, or few, peptides are predicted to have the cit in the P1, P4 or P6 anchor positions. Accordingly, we also predicted the cores for each peptide/allele utilizing NetMHCIIpan substituting native R residues with X. When this was done, only a slight change in attribution was observed, with 6/17 increases for DRB1*01:01, and 6/13 for DRB1*04:01, associated with citrullination of an anchor residue; in the majority of cases, increase in binding were predicted to be with citrullination of a non-anchor residue. Fig 3 summarizes increases in binding affinity as a function of the position of citrullination in the predicted peptide core for binding DRB1*01:01 and DRB1*04:01, as evaluated by both approaches.

Together these results confirm in large set of peptides that the effect of citrullination on peptide binding to two SE+ alleles associated with RA is, in general, minimal. They also reveal that in the majority of instances of increased binding citrullination is predicted at a non-anchor residue. Further, the analysis, which resulted in the identification of 45 citrullinated peptides derived from collagen (highlighted with bold font in S2 Table) that bind HLA-DRB1*01:01 and/or HLA-DRB1*04:01 with an affinity better than the 1000 nM threshold, and thus represent potential targets for additional study, confirms that binding affinity predictions, at least for two SE+ DRB1 alleles, can accurately be approximated using NetMHCIIpan-3.1 using a wildcard representation of citrullinated amino acids.

## Discussion

We report the first comprehensive survey of the effect of citrullination on HLA class II binding, with particular emphasis on two SE-associated alleles. Using four different RA-associated epitopes and a panel of common HLA class II MHC we found that the effects of citrullination on HLA binding are not generalizable, but rather peptide-dependent. In side-by-side experiments with purified HLA, we demonstrate that in some cases citrullinated peptides bind well, but in others they do not. Similarly, compared to the wild-type peptide, in some cases citrullination is detrimental, in others beneficial, and yet in others has no effect.

Binding of citrullinated peptides to HLA-DRB1*01:01 and HLA-DRB1*04:01 was further examined using panels of peptides derived from vimentin and collagen. HLA-DRB1*01:01 and HLA-DRB1*04:01 were chosen because they are represented with high frequency in the general population, are associated with RA, and considered as prototypical SE+ alleles. Furthermore, most *in vivo* studies are performed using HLA-DRB1*01:01 and HLA-DRB1*04:01 transgenic mice, further highlighting the interest of in-depth study of these two alleles. With these peptide panels, representing over 200 citrullinated peptide data points for each allele, no consistent pattern could be associated with citrullination. As with the small set of known epitopes, in some cases, compared to the wild-type peptide, citrullination is detrimental, and in others beneficial, but in the vast majority of cases citrullination has little effect on binding capacity.

Our data support the notion that citrullinated residues at P4 may be beneficial for binding to HLA-DRB1*04:01. Indeed, taken together, increases in DRB1*04:01 and DRB1*01:01 binding with citrullination were more likely to be associated with P4 than other anchor positions. However, it is also apparent that citrullination of P4 did not always lead to increased binding capacity. In some cases the absence of increased binding may be the result of the presence of sub-optimal residues at other main anchors, or deleterious residues at secondary positions, leading to overall low affinity. Thus, it is possible to speculate that fibrinogen 78–91 is a poor SE+ binder, despite the presence of F at the canonical P1 main anchor, because of the presence of residues in other positions (e.g., D at N-1, or N at P3 and P6) that have deleterious effects on binding capacity, thereby mitigating any potential benefit from citrullination. Meanwhile, citrullination of P4 of the vimentin epitope can result in increased DRB1*01:01 and DRB1*04:01 binding because residues in the canonical anchor positions (i.e. V in P1, S in P6 and V in P9) are very well tolerated.

But, our results also challenge the classical view that citrullinated residues are only accommodated within the P4 pockets of HLA-DRB1*01:01 and HLA-DRB1*04:01 by showing that citrullination may be “tolerated” or even beneficial at different positions. In agreement with this data, James et al [56] have described that HLA-DRB1*10:01 presents RA-associated citrullinated peptides by accepting citrulline in three of its binding pockets. Similarly, a recent study by Kampstra et al. showed for several HLA-DQ molecules enhanced affinities for citrulline compared to arginine residues in multiple peptide-binding pockets, including pockets 4, 6, 7, and 9 of HLA-DQ2 and pockets 1, 6, and 9 of HLA-DQ7 and HLA-DQ8 [57]. Further, Roark and co-workers [58], studying the binding of RA associated epitopes, including collagen II 258–272 and vimentin 66–78, for their capacity to bind DRB1*01 subtypes with differential associations with RA susceptibility, identified a role for the MHC B chain residue in position 86, associated with the P1 anchor, in disease susceptibility.

A recent study by Gerstner et al. [59] examined the capacity of native and citrullinated versions of alpha-enolase derived epitopes to bind DRB1*04:01, DRB1*04:04 and DRB1*01:01. Interestingly, in six of the eight cases both versions of the same epitope bound all 3 alleles with similar affinity (i.e., within 3-fold); for one epitope, the citrullinated peptide bound substantially better to two alleles, and for another epitope the citrullinated peptide bound one allele with higher affinity. This overall rate of improved binding (3/24, 12.5%) is not entirely dissimilar from that observed in our analysis. Also in agreement with our results, Gerstner and co-workers found that the citrullinated residues are in various positions, including the canonical P4, P6 and P7 anchors, but also non-anchor positions, to include P-2, P2, P10 and P11; however, in two of the three cases of improved binding citrullination was in P4. Using crystal structures, the same study was able to determine, in the case of one DRB1*04:01 epitope, that T cell specificity was for the citrullinated residue.

Our data also show that citrullination of different positions within the canonical 9-mer MHC binding core does not have a consistent effect on binding capacity. While citrullination at an HLA anchor residue could increase binding capacity in some cases, in the majority of cases of increased binding citrullination was associated with a non-anchor residue. But also, our data show that in the vast majority of cases citrullination, whether at an anchor or non-anchor position, has little effect of binding capacity. Thus, the present results suggest that while novel RA-associated epitopes may result from generation of epitopes with increased binding, it is also likely that a large number of citrullinated epitopes are the result of novel TCR interactions. Together, these data reinforce the need and use of a refined methodology for identifying citrullinated peptide binding beyond the P4 pocket. Using mass spec analyses of peptides eluted from the RA-associated DRB1*01:01, DRB1*04:01 and DRB1*10:01 alleles, as well as non-RA associated alleles, Scholz et al. [60] found some sequence patterns common to peptides bound to the RA-associated alleles, but not present in the non-RA-associated repertoire. However, overall, the degree of repertoire overlap between the alleles studied was found to be a low.

Accordingly, we demonstrated that combination of existing predictive algorithms and *in vitro* testing can identify citrullinated peptides that bind HLA-DRB1*01:01 and HLA-DRB1*04:01 with high affinity, and therefore represent potential epitopes. The performance of this approach was validated using human collagen and vimentin as test cases, and demonstrated that binding of citrullinated peptides could be very accurately predicted using NetMHCIIpan version 3.1 representing citrullinated amino acids as wildcard (“X”).

In this context, our study has identified 117 citrullinated peptides that bind HLA-DRB1*01:01 and/or HLA-DRB1*04:01 with an affinity <1000 nM, a threshold previously identified with the vast majority of HLA class II restricted epitopes [50, 52, 53]. Whether with improved binding or not, compared to the wild-type peptide, these epitopes could be perceived as a new non-self entity by the immune system and thus trigger autoimmune reactions. This study thus represents a large increase in knowledge regarding potential citrullinated epitopes associated with RA. When we examined the citrullinated 1000 nM binders identified herein we found that only 10 overlapped (or were nested with) vimentin and collagen citrullinated epitopes previously described in the literature, as judged by a query of the IEDB on October 2016 [61].

The present study also has potential implications in terms of our understanding of the impact of citrullination on recognition of epitopes in the context of SE alleles. Most studies have focused on modulation of HLA binding, while our study shows that in most cases the effect on binding is rather minor. This suggests that citrullination may be more likely to influence T cell recognition rather than HLA binding. Indeed, it has also been shown that citrullination, depending upon the position it is located, could create a new HLA-DR-restricted functional T cell epitope. In this respect, Chemin et al. have shown that the HLA-DRB1*10:01-restricted citrullinated-CII 311–325 epitope is a neo T cell epitope where citrulline is in position P2, where it would affect T cell recognition [62]; Gerstner et al. [59] have also made similar observations in the context of an alpha-enolase T cell epitope restricted by DRB1*04:01. Thus, the second potential effect of converting arginine to citrulline in a peptide is that it generates a novel TCR interaction site on the peptide when it is bound and presented by the SE+ HLA-DR molecule.

Additional studies will aim at correlating the epitope prediction with the T cell reactivity towards these predicted epitopes in HLA-DRB1*01:01 and/or HLA-DRB1*04:01 RA patients. The determination of the frequencies of predicted citrullinated peptide-specific CD4+ T cells in RA patients (ideally with different disease severity scores versus healthy controls) using MHC II tetramers along with the characterization of the phenotypic (*i*.*e*., memory, naïve, Treg CD4+ T cells) and functional (*i*.*e*., cytokine profile produced upon antigen stimulation) nature of the responding T cell repertoire will help to further highlight the relevance of this methodology in regard to RA pathogenesis [16–18, 35].

## Supporting information

S1 Table Panel of HLA class II alleles studied. (PDF)

S2 Table Vimentin and collagen MHC-peptide binding data and predicted cores. (XLSX)

S3 Table Effect on binding capacity as a function of the predicted position of citrullination in relationship to the MHC-binding core. (PDF)
