# Supplementary material for: Citrullination only infrequently impacts peptide binding to HLA class II MHC
Source: PLoS One. 2017 May 8;12(5):e0177140. doi: 10.1371/journal.pone.0177140 (PMC5421785; doi:10.1371/journal.pone.0177140)
Supplement: S1 Table — (PDF) [file pone.0177140.s001.pdf]

Supplemental Table 1

Locus	Alpha chain	Beta chain	Population frequency	SE nomenclature (sequence) - RA association [odds ratio]
**DR**	DRA1*0101	DRB1*0402	2.2	S1 (ARAA or ERAA) - protective allele [0.39]
	DRA1*0101	DRB1*1302	7.7	S1 (ARAA or ERAA) - protective allele [0.39]
	DRA1*0101	DRB1*1501	12.2	S1 (ARAA or ERAA) - protective allele [0.39]
	DRA1*0101	DRB1*0401	4.6	S2 (KRAA) – predisposing allele [3.21]
	DRA1*0101	DRB1*0101	5.4	S3P (Q/R RRAA) – predisposing allele [1.61]
	DRA1*0101	DRB1*0404	3.8	S3P (Q/R RRAA) – predisposing allele [1.61]
	DRA1*0101	DRB1*0405	6.2	S3P (Q/R RRAA) – predisposing allele [1.61]
	DRA1*0101	DRB1*1001	3.8	S3P (Q/R RRAA) – predisposing allele [1.61]
	DRA1*0101	DRB1*1101	11.8	S3D (DRRAA) – protective allele [0.37]
	DRA1*0101	DRB1*1201	3.9	S3D (DRRAA) – protective allele [0.37]
	DRA1*0101	DRB1*1602	7.7	S3D (DRRAA) – protective allele [0.37]
	DRA1*0101	DRB1*0301	13.7	X (non-RAA motif) – non-carrier [1]
	DRA1*0101	DRB1*0701	13.5	X (non-RAA motif) – non-carrier [1]
	DRA1*0101	DRB1*0802	4.9	X (non-RAA motif) – non-carrier [1]
	DRA1*0101	DRB1*0901	6.2	X (non-RAA motif) – non-carrier [1]
**DRB3/4/5**	DRA1*0101	DRB3*0101	26.1	
	DRA1*0101	DRB3*0202	34.3	
	DRA1*0101	DRB4*0101	41.8	
	DRA1*0101	DRB5*0101	16.0	
**DP**	DPA1*0103	DPB1*0201	17.5	
	DPA1*0103	DPB1*0401	36.2	
	DPA1*0103	DPB1*0402	41.6	
	DPA1*0202	DPB1*0501	21.7	
**DQ**	DQA1*0102	DQB1*0602	14.6	
	DQA1*0501	DQB1*0301	35.1	
	DQA1*0301	DQB1*0302	19.0	
	DQA1*0501	DQB1*0201	11.3	
	DQA1*0101	DQB1*0501	14.6	
