# Supplementary material for: Citrullination only infrequently impacts peptide binding to HLA class II MHC
Source: PLoS One. 2017 May 8;12(5):e0177140. doi: 10.1371/journal.pone.0177140 (PMC5421785; doi:10.1371/journal.pone.0177140)
Supplement: S3 Table — (PDF) [file pone.0177140.s003.pdf]

Supplemental Table 3

WT sequence predicted core								
	DRB1*01:01				DRB1*04:01			
Position	Increase	Neutral	Decrease	Total	Increase	Neutral	Decrease	Total
-6	0	1	0	1	0	1	0	1
-5	0	3	0	3	0	4	0	4
-4	0	2	1	3	0	3	0	3
-3	0	7	0	7	0	5	0	5
-2	1	14	6	21	0	17	2	19
-1	0	11	0	11	1	8	1	10
1	0	1	0	1	0	3	0	3
2	4	21	8	33	3	33	2	38
3	0	12	3	15	1	10	2	13
4	1	1	1	3	0	0	0	0
5	1	13	0	14	2	12	0	14
6	0	0	0	0	0	2	0	2
7	3	13	2	18	2	18	0	20
8	4	16	4	24	1	20	1	22
9	0	4	1	5	2	7	1	10
10	1	16	0	17	0	16	0	16
11	0	9	1	10	0	7	1	8
12	2	4	2	8	1	7	0	8
13	0	7	0	7	0	7	0	7
14	0	2	1	3	0	2	0	2
15	0	2	0	2	0	1	0	1
Anchor	4	19	4	27	4	30	1	35
Non-anchor	13	140	26	179	9	153	9	171
Total	17	159	30	206	13	183	10	206

Cit ("X") sequence predicted core								
	DRB1*01:01				DRB1*04:01			
Position	Increase	Neutral	Decrease	Total	Increase	Neutral	Decrease	Total
-6	0	0	0	0	0	1	0	1
-5	0	3	0	3	0	5	0	5
-4	0	2	1	3	0	3	0	3
-3	0	6	0	6	0	3	0	3
-2	1	12	6	19	0	17	2	19
-1	0	13	0	13	1	9	1	11
1	0	10	1	11	0	9	1	10
2	3	12	6	21	2	23	1	26
3	0	10	3	13	1	9	2	12
4	3	5	1	9	2	7	0	9
5	1	11	0	12	1	11	0	12
6	1	4	1	6	0	4	0	4
7	2	12	2	16	2	17	0	19
8	3	17	4	24	1	18	1	20
9	0	6	1	7	2	6	0	8
10	1	12	0	13	0	16	0	16
11	0	8	1	9	0	7	1	8
12	2	4	2	8	1	8	0	9
13	0	8	0	8	0	7	0	7
14	0	2	1	3	0	2	1	3
15	0	2	0	2	0	1	0	1
Anchor	6	37	6	49	6	43	1	50
Non-anchor	11	122	24	157	7	140	9	156
Total	17	159	30	206	13	183	10	206
